# An Ecological Approach to Disaster Mitigation: A Literature Review

**DOI:** 10.7759/cureus.45500

**Published:** 2023-09-18

**Authors:** Rebecca Pratiti

**Affiliations:** 1 Internal Medicine, McLaren Health Care Flint, Flint, USA

**Keywords:** health effects, economic factors, disaster mitigation, disaster effects, disaster preparedness, vulnerability index, disaster recovery

## Abstract

Disasters, whether natural or manmade, disrupt the functioning of communities, significantly impacting people’s lives and health. To build community resilience, the Centers for Disease Control and Prevention recommends community preparedness, where multiple stakeholders work together. Disaster Preparedness Science Research (DPSR) similarly encourages the improvement of disaster relief outcomes. This literature review assesses the vulnerability of communities for prioritized intervention, summarizes disaster effects, and suggests the scope for improvement in disaster preparedness (DP). Twenty-one articles were reviewed based on disaster mitigation and economic factors from 90 studies identified through a PubMed search till September 2021. Vulnerable communities with higher hazard risks are identified by vulnerability indices (VI), including the Climate Risk Index, Environmental VI, and Socio-Economic VI. However, VI predicting one disaster may not predict another. Disaster behavioral response involves five phases. Disaster effects include medical, mental, environmental, and economic effects, as well as the unique recovery time from each domain effect. Medical effects include malnutrition, malaria, diarrhea, heat stress, exacerbations of chronic conditions, infectious disease outbreaks, trauma, and death. Mental effects are post-traumatic stress disorders, depression, anxiety, somatic complaints, psychological distress, sleep problems, and suicides. Environmental effects include isolation, migration, injury to family members, life threats, and property damage. Loss of livelihood and property are associated with worse outcomes. Disaster recovery, which is seldom measured and not clearly defined, affects measurement and comparison across settings. A uniform validated VI, including multiple indicators assessing vulnerability to various disasters, is required. Livelihood restoration is integral to mental health recovery in some disaster types. Fund diversification, prioritized to the vulnerable and to each domain effect of disaster in the immediate post-disaster phase, expedites recovery. Later recovery investments focused on helping people rebuild their community enhance psychological outcomes. Promoting job insurance in highly vulnerable labor-based communities with high VI, wherein willing-to-pay is high, could facilitate faster recovery. DPSR should be encouraged.

## Introduction and background

The National Disaster Life Support Foundation defines a disaster as ‘an event and its consequences that result in a serious disruption of the functioning of a community and cause widespread human, material, economic, or environmental losses that exceed the capacity of the affected area to respond to without external assistance to save lives, preserve property, and maintain the stability and integrity of the affected area’ [1]. Pollution can directly cause environmental contamination and adverse health effects, as well as indirectly contribute to climate change, which can increase the risk of disasters. Thus, climate risk assessment is an essential part of the Intergovernmental Panel on Climate Change’s (IPCC) review assessment [2]. From 1980 to 2014, the United States experienced 178 natural disasters with an estimated cost of 1 trillion dollars, and between 2000 and 2012, approximately 1.2 million people died worldwide, with an estimated 1.7 trillion dollars in damages. Besides these enormous economic costs, disasters also disrupt the lives of individuals, families, and communities at multiple levels [3]. According to the IPCC, the severity of climate disasters depends on a community’s vulnerability to exposure and the rates of exposure. Vulnerabilities are determined by economic, social, geographic, demographic, cultural, institutional, governance, and environmental factors that vary dynamically across temporal and spatial scales. These vulnerabilities can be modified by developing resilience and adaptive mechanisms for coping within exposed communities. The major challenge, however, is making prioritized decisions with risks and benefits [4]. The Centers for Disease Control and Prevention’s (CDC) 2011 core public health preparedness measures included community preparedness, which entails community engagement and partnership development. The Federal Emergency and Management Agency’s (FEMA) “whole community approach” framework for emergency management equally involves multiple stakeholders, including government and community leaders, working together to build community resilience [5]. 

Disaster Preparedness Science Research (DPSR) is encouraged to improve disaster relief outcomes. This literature review assesses the vulnerability of communities for prioritized intervention, summarizes disaster effects, and suggests ways of improving disaster preparedness. Twenty-one articles on disaster mitigation and economic factors from 90 studies identified through a PubMed search term ‘disaster mitigation economic factors’ up to September 1st, 2021, with English articles only being used in the review. The abstract of the review was presented virtually at the 8th International Conference on Public Health (ICOPH 2022) on 29th July 2022.

## Review

Risk assessment by risk indicators

Disaster risk refers to the possibility of future adverse effects to which exposure to hazards and the vulnerability of the exposed contribute, while vulnerability refers to the probability of humans experiencing the effects of hazards. Vulnerability to one hazard is considered to be independent of vulnerability to other hazards. For example, vulnerability to a financial crisis does not imply vulnerability to climate change or natural hazards, and a population vulnerable to hurricanes or floods may be invulnerable to landslides [4]. The household vulnerability index may contribute to vulnerability of a community but could not be used as a proxy for a community vulnerability for disasters. It may be used as an indicator for resource allocation. Household vulnerability is further subdivided into financial and social vulnerability. How it is measured varied widely across different countries and hence has not been evaluated further as a part of disaster risk assessment [6,7]. Vulnerability factors present in a community are not necessarily created by a disaster but are rather revealed during a disaster, although an abnormal development, such as unplanned urbanization or inadequate job diversification, can make a population vulnerable [8,9]. Some risk indicators, such as those used in quantitative vulnerability research, give a more accurate risk assessment, depending on the research question, location of the study, and the researcher’s individual judgment. [10]. Some of the recent risk indices are described below.

Climate Risk Index

Climate Risk Index (CRI) can assist national authorities in formulating adaptation policies and plans by identifying key threats, issues, and vulnerabilities in the initial problem formulation phase [11,12]. It can serve as a quick screening tool to identify and prioritize regions or provinces with a greater likelihood of being adversely affected by climate extremes [2]. The CRI combines (i) climate change-amplified hazards with selected extreme climate indices, (ii) indicators of exposure of key economic, social, natural, and manufactured capital assets, and (iii) vulnerability, which comprises sensitivity to climate-induced hazards and adaptive capacity. The CRI allows for sub-national climate adaptation planning and needs assessment. Extreme climate indices (CEI) used in the study include heat wave magnitude, cold wave magnitude, heavy rain days, and consecutive dry days. Exposure indicators included infrastructure density, urban areas, industrial areas, and structural dependency index [2].

Environmental Vulnerability Index

Grigorescu et al. developed a theoretical framework for constructing and computing the Environmental Vulnerability Index (EVI) by identifying three aspects of community vulnerability, namely ecosystem integrity or degradation, risk, and resilience factors. These vulnerabilities can be measured using a variety of indicators such as temperature, waste, vegetation, land cover, land use, soil degradation, precipitation, population density, etc. EVI was integrated with a socio-economic vulnerability index (SEVI) to develop a heat vulnerability index (HVI) [13]. 

Socio-Economic Vulnerability Index

SEVI offers a wide variety of criteria for measuring community factors such as the quality of human settlements (e.g., housing type and construction, infrastructure), medical services, employment loss, rural/urban proportion, education, etc. It also measures population characteristics such as age, gender, race, ethnicity, and socioeconomic status as determined by income [13-16]. 

Palmer Drought Severity Index

Palmer Drought Severity Index (PDSI), one of the oldest and most used indeces, predicts drought occurrence and resolution of established drought. It is calculated using precipitation, temperature and soil moisture data. It is widely used to detect agricultural drought. PDSI may lag emerging droughts and it is not effective for mountainous areas with frequent climatic extremes or during winter and spring. Some other drought indices that are used include standard precipitation index, percent of normal precipitation and normalized difference vegetation index. These indices could only predict drought and not other disasters. Further, since these indices only include meteorological data without community data, they could not predict the effect of droughts in a particular community or region [17].

Effects of disaster 

Most natural disasters are sudden occurrences that affect people’s lives and health significantly. There has been considerable progress and expansion towards a better understanding of such impacts of disasters on human beings in the last few years [18]. While some disasters directly affect human lives and health, others have a more economic effect. Despite a reduction in the number of people being killed in disasters, the number of affected people is on the rise [19]. Pasnau and Fawzy (1989) identified five phases in the behavioral and psychological responses to natural disasters: (1) The impact phase during the onset of the disaster, characterized by fear and confusion; (2) The heroism (or rescue) phase, characterized by altruistic interventions from humanitarian organizations; (3) The honeymoon (or remedy) phase, characterized by active community collaborations with a sense of renewed hope for the collective good; (4) The disillusionment phase, characterized by disappointment and a sense of distress at a seemingly unjust resource allocation, potentially triggering mental health problems; (5) The reorganization (or reconstruction and recovery) phase, characterized by people starting to rebuild and depend on themselves again. This last phase is important and failing it may cause long-term animosity and bitterness [20]. Although this model is from 1989, it still seems relevant to disaster response. It is important, therefore, to identify factors and processes that facilitate resilience and positive adaptation of individuals using an ecological framework [3]. This cycle is different from the World Health Organization (WHO) disaster cycle since it is more pertinent to how the community reacts as compared to how disaster relief is implemented. The disaster cycle involves four phases: preparation, response, recovery, and mitigation. The cycle illustrates the steps that emergency managers take when planning for and responding to a disaster. Preparation is the phase where response plans are constructed. The response is the phase where there is immediate action to limit the hazards created by the disaster. Recovery is the effort to return a community to pre-disaster levels of functioning. Mitigation is the phase where new measures are undertaken to prevent or minimize the effects of future disasters. Some sources refer to the mitigation phase as “prevention” [1].

Medical Conditions

Disasters affect human well-being and health in many direct and indirect ways. It could include the effect on physical health including trauma (e.g., with floods, droughts). Between 2030 and 2050, approximately 250,000 additional global deaths are expected to occur annually due to malnutrition, malaria, diarrhea, and heat stress resulting from climate change effects [21]. Disasters cause outbreaks of infectious diseases, exacerbations of chronic diseases, and malnutrition [22,23]. Heat waves can cause heat-related illnesses [24, 25]. Health effects could also be caused by exposure to hazardous physical, chemical and biological agents in air, water, soil and food. Extreme weather events are also causing multiple health problems including worsening of heart or lung conditions [18].

Mental Conditions

The mental health impacts of disasters often include post-traumatic stress disorders (PTSD), depressive or anxiety disorders, somatic complaints, and general mental morbidity [26]. Short-term exposure to disasters can trigger psychological distress, somatic complaints, sleep problems, and psychosocial and behavioral problems [3]. Most of these effects are mild to moderate and only about 30% of symptomatic cases require intervention. Elderly people with high rates of chronic illnesses are especially susceptible to the psychological and physical stresses of disasters [27]. Mental health impacts are worse for women and people with a low socioeconomic status as measured by education or income. Mental health recovery has been shown to be mainly a function of time [26]. Disasters have been linked to increased suicide rates that may take up to two years to recover to baseline [28]. The risk of behavioral problems and suicide remains high in affected communities many years after a disaster, highlighting the need for sustained post-disaster surveillance [19].

Environmental Conditions

Universal environmental risk factors that influence the severity of individual exposure include bereavement, injury to oneself or family members, life threats, panic, and extensive loss of property [26]. The impact of disasters varies significantly between regions due to differences in their social, economic, and environmental characteristics. Urban areas are more vulnerable to heat stress effects, including heat waves and their health effects, while rural areas are more vulnerable to drought and its impact on agriculture. A community’s resilience to a given disaster also depends on its natural and built environment. Appropriate land use, crop production, water resources or forest ecosystems can predict disaster exposure risk [13,29,30]. There is a significant relationship between the Human Development Index and disaster-related mortality [18]. Food insecurity can worsen after a disaster. At the community level, disaster-related damage to infrastructure can disrupt normal community functioning by way of temporary school closures and reduction in the availability of critical commodities such as food, water or electricity [31]. Disasters lead to social isolation, out-migration, and separation from families [32]. When people are displaced or their known environments are altered by a disaster, the material and cultural resources on which their lives depended as individuals and as a community are separated from them, triggering distress and upsetting the networks of social relationships [4]. A post-typhoon survey to identify outcome indicators of populations functioning poorly and likely in need of services found that 65-plus-year-olds living outside their own homes and working, with a moderate or poor health condition were the most relevant risk factors of disability at six months after the typhoon [33].

Economic Conditions

Adverse outcomes are associated with loss of livelihood and property such that restoring livelihood is an important aspect of mental health recovery [34]. Bankruptcy and the ratio of effective job offers were not significantly associated with post-disaster suicide rates in the case of an earthquake [28], although households with an unemployed head and those that had lost their fishing boats had lower odds of recovery in the case of a tsunami [35]. Widespread damage to communities and the resulting economic impact interact with other factors, including death and illness, to worsen post-disaster social vulnerability [36]. Though most funding in the post-disaster phase comes from governments, other funding options include non-governmental organizations, family, and religious organizations. Thus, diversifying funding in the immediate post-disaster phase is important for better recovery. Financial capital, common among high-income groups, is associated with successful economic recovery [35]. The European Commission has developed a set of indicators to evaluate adaptation efforts and vulnerabilities in the form of sensitivity to disaster, exposure, and coping capacity at a national level. Some of these indicators are demographic size, population density, the share of elderly aged over 65, and the share of people under five, blue areas, water supply, impervious areas, green areas, air conditioning, and sewage [37].

Scope for improvement

Disaster Preparedness Funding

It is important to define recovery in the setting of disaster to be able to measure and compare it across various settings of regions and countries. For example, full recovery is defined as when the community has returned to its pre-disaster functional level. Immediate recovery could be defined where 50-60% of the people have returned to the pre-disaster functionality. Recovery can be physical, emotional, mental, or financial in the form of livelihood or assets. Recovery goals can be set, aiming to recover one-third of pre-disaster status with the ultimate goal of achieving full recovery. During the initial stage of one-third recovery (relief phase), funds can be invested directly at the individual level, while eventual recovery investments focus on helping the community rebuild its assets. This has been shown to be a psychological intervention and recovery path for communities. Post-disaster fund-diversifying policies may also be integrated into disaster planning. Infrastructure-based communities will likely invest more in infrastructure insurance, while labor-based communities will be more inclined toward job insurance. Some insurance schemes have disability options that cover physical problems a person may face during a disaster. However, losing one’s livelihood even without any physical loss remains a major stressor. Short-term job insurance for three to six months may help families recover irrespective of available funds. This immediate relief fund option is important since the amount and type of public health preparedness and response federal funding varies for disasters [38]. A community’s willingness to pay for a disaster-related infrastructure or job insurance depends upon its disaster vulnerability risk. This approach also helps to focus funds on people with a permanent disability, the elderly, and families with losses or low economic status.

Validated Disaster Preparedness Index

Developing a vulnerability index with good sensitivity and specificity for identifying or predicting an adverse event or disaster is a cumbersome process. Most disaster indices use meteorological, agricultural, hydrological, or socioeconomic indicators, with few utilizing an ecological approach to predict vulnerability at a subnational level. Determining the appropriate geographical unit for a vulnerability index is challenging, as a broader geographical area may neglect vulnerable regions, while a localized approach may delay timely response. A modified approach is to evaluate localized areas and compare the cumulative vulnerability risk within a larger geographical area [18]. However, vulnerability indices designed for one disaster, such as heat-related events, may not predict vulnerabilities for other disasters, such as floods. Therefore, multiple indicators for extreme weather events may be necessary for a comprehensive vulnerability index. Other useful indicators include dependency ratio, part-time to full-time employment ratio, unemployment rate, the ratio of industrial-based to office-based jobs, and availability and accessibility of health services. Ultimately, the selection of indicators for any composite index is influenced by the aim of the study, policy implications and the availability and accuracy of statistical data for the analyzed phenomena [13].

Other Factors

Data generated via disaster citizen science mitigates the adverse impacts of disaster, building social networks, and developing skills that empower communities [5]. Many factors motivate disaster preparedness behavior, including past experiences, social media, and a sense of responsibility for one’s own safety [39]. Some non-risk-based disaster preparedness interventions include improving climate literacy and early warning systems. Public health measures to prevent adverse health impacts from disasters include surveillance and control of infectious diseases, specifically improving access to improved sanitation, safe water, food security, and solid waste management. Managing critical infrastructure, including healthcare, mental health services, safe shelters to prevent or mitigate displacement, and effective warning and informing systems [4] is also crucial. The most commonly reported outcomes of any disaster are often limited to the number of people killed and economic damage, with little or no attention given to the long-term disability or well-being of affected populations in terms of disability-adjusted life years (DALY) or quality-adjusted life years (QALY). This is due to the difficulty of measuring these units in relation to a disaster, highlighting the need for further surveillance of long-term health outcomes of disasters [33]. Adding quantitative assessments for disaster recovery may possibly make it easier to measure. For example, percentage population density regained, and percentage average household income regained could help further assess and intervene during the mitigation and prevention phase. Further implementing community resiliency model goal is to help create “trauma-informed” and “resiliency-focused” communities that share a common understanding of the impact of trauma and chronic stress on the nervous system and how resiliency can be restored or increased using this skills-based approach [40].

Limitation

Though a healthy ecosystem is a natural barrier to disaster, this review did not evaluate this approach to disaster mitigation such as planning of activities for ecosystem restoration, integration of risk reduction in ecosystem development planning and post-disaster strategies. DPSR is a wide topic including multiple ways to improve disaster preparedness and mitigation. The scope of the review was focusing on the economic factor. There are financial implications in developing a healthy ecosystem, further studies are needed to understand if specific allocated funds utilized for better ecosystem planning and development are cost-efficient. We only reviewed studies from PubMed. 

## Conclusions

It is crucial to adopt an ecological perspective for disaster mitigation and to understand the impact of different disaster types on physical, mental, environmental, and economic well-being at both individual and community levels. Recovery from disasters is often not measured, and there is a lack of clear definition of recovery, making it difficult to compare and measure across different settings. Developing a validated vulnerability index that includes multiple indicators assessing vulnerability to various disasters is necessary.

Restoring livelihoods is an essential part of mental health recovery in some disaster types. It is important to diversify funds in the immediate post-disaster phase and prioritize for known vulnerable domains, which can expedite recovery. Later investments should focus on helping people build their communities, which can enhance psychological outcomes. In highly vulnerable labor-based communities with high VI, where the willingness to pay is high, promoting job insurance could facilitate faster recovery. Scientific research on disaster preparedness should be encouraged.
